# Influence of the Production System (Intensive vs. Extensive) at Farm Level on Proximate Composition and Volatile Compounds of Portuguese Lamb Meat

**DOI:** 10.3390/foods10071450

**Published:** 2021-06-22

**Authors:** Noemí Echegaray, Rubén Domínguez, Vasco A. P. Cadavez, Roberto Bermúdez, Laura Purriños, Ursula Gonzales-Barron, Ettiene Hoffman, José M. Lorenzo

**Affiliations:** 1 Centro Tecnológico de la Carne de Galicia, Avd. Galicia No 4, Parque Tecnológico de Galicia, San Cibrao das Viñas, 32900 Ourense, Spain; noemiechegaray@ceteca.net (N.E.); robertobermudez@ceteca.net (R.B.); laurapurrinos@ceteca.net (L.P.); jmlorenzo@ceteca.net (J.M.L.); 2 Centro de Investigação de Montanha (CIMO), Instituto Politécnico de Bragança, 5300-253 Bragança, Portugal; vcadavez@ipb.pt (V.A.P.C.); ubarron@ipb.pt (U.G.-B.); 3 Faculty of Management, Canadian University Dubai, Dubai 117781, United Arab Emirates; ettiene.hoffman@cud.ac.ae; 4 Área de Tecnología de los Alimentos, Facultad de Ciencias de Ourense, Universidad de Vigo, 32004 Ourense, Spain

**Keywords:** Bordaleira-de-Entre-Douro-e-Minho, rearing system, pasture, concentrate, volatile compounds

## Abstract

Today’s society demands healthy meat with a special emphasis on integrated animal husbandry combined with the concern for animal welfare. In this sense, the raising of lambs in an extensive system has been one of the most common practices, which results in meats with high nutritional value. However, both the production system and the diet play a fundamental role in the chemical composition of the meat, which has a direct impact on the content of volatile compounds. Thus, the aim of this study was to determine the effect of two production systems (intensive and extensive) on the chemical composition and volatile profile of lamb meat. Twenty-eight lambs of the Bordaleira-de-Entre-Douro-e-Minho (BEDM) sheep breed were raised for meat production under the intensive or extensive system and were fed with concentrate and pasture, respectively. All animals were carried out in the muscle *longissimus thoracis et lumborum*. Results evidenced that all the composition parameters were affected by the production system. Extensively-reared lambs produced meat with the highest fat and protein contents, while these animals had the lowest percentages of moisture and ash. Similarly, the total content of volatile compounds was affected (*p* < 0.05) by the production system and were higher in the meat of lambs reared extensively. Furthermore, the content of total acids, alcohols, aldehydes, esters, ethers, furans and sulfur compounds as well as most of the individual compounds were also affected (*p* < 0.05) by the production system, whereas total hydrocarbons and ketones were not affected (*p* > 0.05). As a general conclusion, the production system had very high influence not only in proximate composition but also in the volatile compounds.

## 1. Introduction

The meat quality is an essential factor in ensuring consumer satisfaction [1] and is related to several parameters such as visual appearance, quality and distribution of the fat, texture, juiciness as well as flavor [2]. Specifically, in lamb meat, the odor and flavor are two of the most important eating quality attributes since the meat of these animals have a unique aroma [3,4,5]. In this manner, lamb meat is characterized by a typical species-related flavor that is denominated as “mutton flavor”, which could seriously affect the acceptability of consumers [6,7].

On the other hand, in response to consumer demand, the sheep farming sector is increasingly concerned with incrementing the added value of its products through sustainability, animal welfare and conservation of ancient autochthonous genetic types [8,9]. In this regard, the use of autochthonous breeds for meat production is of special interest due to the promotion of the valorization, protection and conservation of the zoogenetic heritage [10]. This is the case of the Portuguese native breed, named Bordaleira-de-Entre-Douro-e-Minho (BEDM), which can also contribute to the diversity of production systems due to its particular characteristics such as local adaptation, resistance to diseases and high fertility [11,12]. These qualities allow the use of natural pastures in lamb rearing [13]. Nevertheless, the characteristics generated by extensive rearing can sometimes result in various unwanted modifications in the organoleptic quality of the lamb meat with respect to intensive commercial farming. This is the case of the volatile profile, which in addition to being influenced by the animal’s genetics, slaughter age and management practices is strongly influenced by the diet supplied [6,14,15]. In fact, previous studies have linked certain volatile compounds with a specific diet [16,17]. Thus, volatile substances such as terpenoids [14,18], phenols [19] and the diketone 2,3-octanedione [14,18,20] were related with pasture-based diets; while lactones [20,21], branched fatty acids [6,20,22] and compounds such as 2,3-butanedione [23] and furan, 2-pentyl [24] have been linked to grain-based diets.

Therefore, the overall purpose of the present experiment was to evaluate the influence of the production system (intensive and extensive) on the chemical composition and the volatile profile in the muscle *longissimus thoracis et lumborum* of BEDM breed lambs.

## 2. Materials and Methods

### 2.1. Lamb Rearing and Feeding

In the present study, 28 lambs (males) of the Bordaleira-de-Entre-Douro-e-Minho (BEDM) sheep breed were raised for meat production in the Atlantic bioregion of Ponte de Lima (at Ponte Lima Agrarian School) under two different exploitation regimes: intensive and extensive system. Lambs were randomly selected from the flock and all of them were born and raised single. The weight of the lambs reared in the intensive production system at birth was 2.57 ± 0.28 kg, while those reared in the extensive regiment was 2.45 ± 0.27 kg, with no significant differences (*p* = 0.278) in the initial weights at the beginning of the experiment. In both farms, the feeding system was based on semi-natural pastures improved by sowing perennial ryegrass (*Lolium perenne*). The pastures were mainly constituted by grasses (54.3%) and legumes (28.9%). Specifically, 15 BEDM lambs were reared in the fall of 2018 under the intensive system and 13 BEDM lambs were reared in the spring of 2019 in the extensive system. Animals reared under the intensive system remained with the mothers and had ad libitum access to natural grass hay from birth to 3 months of age. After weaning (3 months), the lambs continued to be fed natural grass hay, in addition to 300 g/day of commercial compound feed supplied in two intakes per day (9:00 a.m. and 5:00 p.m.). The commercial compound feed used in the diet of intensively-reared lambs of the present research was supplied by Alimentação Animal Nanta S.A. (Marco de Canaveses, Portugal) and it was composed (in unknown proportions) of barley, wheat bran, extruded dehulled soy meal, dry beet pulp, brewers’ dried grains, soy hulls, beet molasses, wheat germ, calcium carbonate, sunflower seed meal (extracted), soy oil, sodium chloride and a vitamins and minerals mix. Its chemical composition was the following: protein: 15.5%; ether extract: 4.5%; fiber: 8.2%; ash: 8.2%; calcium: 1.1%; phosphorous: 0.40%; sodium: 0.37%. All the information on the composition and ingredients of the commercial compound feed can be found in Supplementary Table S1. On the other hand, the lambs reared under the extensive system had access to their mother’s milk (unweaned) and they went out to graze (ad libitum) with the herd from morning until dark during the entire experiment (from birth to slaughter; about 4 months). Upon darkness, the lambs were sheltered in stables where they also had access to meadow hay and water ad libitum. The growth test was carried out for 4 months and so the phenological status of the pasture was very varied. This test aimed to characterize the production systems in a holistic perspective; thus, the animals’ feed was the one usually used in the farms.

### 2.2. Lamb Meat Samples

The trial planned to slaughter the animals at 4 months of age. Thus, the age at slaughter varied between 4 and 4.5 months and the births were not synchronized, which translated into the variation in the age at slaughter. With this in mind, at 4–4.5 months old, the lambs were transported to a commercial abattoir of Portugal. The animals were handled in batches ranging from 5 to 12 lambs and they were slaughtered according to the conditions previously reported [9]. Lambs reared in an intensive production system had a live weight of 13.54 ± 1.48 Kg (5.93 ± 1.02 Kg hot carcass weight), while those reared in an extensive production system had a live weight of 12.44 ± 2.65 kg (8.29 ± 1.77 Kg hot carcass weight). The live weight between both groups did not show significant differences (*p* = 0.176, while carcass weight of extensively-reared animals was significantly higher (*p* < 0.01) than those reared in the intensive production system. After cooling, the *longissimus thoracis et lumborum* muscles were removed from the sixth to the thirteenth vertebrae of lamb carcasses (a total of 56 pieces, 2 muscles, left and right and for each carcass). All muscle pieces were vacuum packed, refrigerated and transported to the CTC lab for the analysis. The left side was used for proximate composition analysis (72 h post-slaughter), while volatile analysis were carried out in the right muscle after refrigerated storage (4 ± 1 °C for 15 days). Before the analysis, a steak of each muscle (about 80 g) was conveniently chopped and homogenized in order to obtain a representative sample of each animal.

### 2.3. Analysis of Chemical Composition

Moisture [25], protein (Kjeldahl *N* × 6.25) [26] and ash [27] were determined and expressed as percentage following the ISO recommended standards, while intramuscular fat was quantified according to the American Oil Chemistry Society (AOCS) official procedure [28].

### 2.4. Volatile Compounds Analysis

For the volatile compound analysis, Headspace-Solid phase microextraction (HS-SPME) technique was used for the volatile extraction and concentration, while the separation and identification of each volatile was carried out using gas chromatography coupled with mass spectrometry (GC-MS) (Agilent Technologies, Santa Clara, CA, USA) equipped with the DB-624 capillary column (30 m, 250 μm i.d., 1.4 μm film thickness; J&W Scientific, Folsom, CA, USA). All analysis steps, chromatographic and mass spectrometer conditions and data processing were previously reported [29]. The results were expressed as area units of the extracted ion chromatogram from the quantifier ion (*m*/*z*) per gram of sample (AU × 10^4^/g of sample). The Linear Retention Index (LRI) was calculated for the aforementioned capillary column (DB-624). Both LRI and *m*/*z* values are presented in all volatile tables as additional information to the volatile analysis.

### 2.5. Statistical Analysis

A total of 56 samples (28 for the chemical analysis and 28 for the volatile compounds determination) were analyzed in triplicate for each parameter. Normal distribution and variance homogeneity had been previously tested (Shapiro-Wilk). The influence of the production system on the chemical composition and volatile compounds was evaluated with one-way analysis of variance (one-way ANOVA) using the SPSS package version 23.0 (IBM SPSS, Chicago, IL, USA). Significant differences were indicated at *p* < 0.05, *p* < 0.01 and *p* < 0.001. Furthermore, the Pearson’s linear coefficient was employed to determine correlations between the intramuscular fat and volatile content using the same statistical software.

## 3. Results and Discussion

### 3.1. Chemical Composition

The proximate composition of the BEDM lamb meat from the different production systems is shown as percentage in Table 1.

The values found for the proximate composition parameters agree with those reported by other authors. In this regard, a recent study comparing three different lamb breeds found values for fat (about 1.6%), protein (19–21%), moisture (75–77%) and ash (1.06–1.22%) and are similar to those described in this study [30]. Similarly, an investigation studying the influence of five different breeds and three (intensive, semi-extensive and extensive) production systems [9] or the influence of different slaughtered ages also showed comparable values for all proximate parameters.

As it can be observed, the production system significantly (*p* < 0.001) affected all the composition parameters. Concretely, the extensive production system provided lambs with a significantly (*p* < 0.001) higher intramuscular fat (IMF) and protein content than the intensive production system (1.51 vs. 0.49% and 20.92% vs. 19.32%, respectively). In contrast, intensively-reared lambs showed significantly (*p* < 0.001) higher amounts of moisture and ash (78.00 vs. 75.91% and 1.37 vs. 1.20%, respectively). Our results agree with those reported by other authors who observed that lamb meat with the highest moisture content presented the lowest IMF and protein contents [31]. Thus, inverse correlation between the moisture and IMF contents previously described in the lamb meat [9,31] explain our findings. However, these differences do not remain constant throughout the literature. Other studies found that grass-fed lambs decreased intramuscular fat [32,33] and protein content [34] while the moisture percentage was increased [34]. Several authors even observed that not all composition parameters were affected by the diet [35,36,37]. Among all proximate composition parameters, intramuscular fat is an important parameter that influenced the lamb meat quality. However, there is controversy about the influence of multiple factors on this content. In this regard, a recent study demonstrated that rearing season had an important effect on IMF content [38]. Sheep reared in spring presented higher IMF content than those reared in autumn. This fact could partially explain the results obtained by us, since the lambs reared in the extensive system (spring of 2019) presented higher values of IMF than those reared in the intensive system (fall of 2018). The differences in the availability and the quality of pasture could be an important factor that could explain the fact that animals reared in spring presented higher IMF than those reared during the autumn, since the two main peaks in lamb feeding change are in winter and spring [38]. Additionally, the better quality of the pasture also results in a better milk production by the mothers characterized by a high fat content due to a diet rich in fiber, which is undoubtedly related to the higher IMF content in the animals raised in the extensive system (unweaned) than those raised in the intensive production system (weaned). In line with the aforementioned elements, another important factor that influences IMF content is the diet. Generally speaking, the lambs feeding with concentrate presented higher IMF than those feeding with pasture or silage. This fact was corroborated by Cadavez et al. [9], who reported that the lambs reared in intensive production systems had higher IMF than those reared in semi-extensive or extensive systems. This is related with the fact that feedlot lambs had lower energy expenditure for grazing than lambs reared in the extensive system [39]. However, as reported in the Material and Methods section, in the present study both groups of animals graze and, thus, in our study we expect similar expenditure for grazing in animals from both production systems. Contrary to the results reported by Cadavez et al. [9], a study in which lambs received silage, silage + concentrate or concentrate during 36, 54 or 72 days concluded that both diet and feeding durations did not have an effect on IMF [34]. They attributed the lack of differences to the similarity in energy expenditure between animals and a higher rate of gain from good quality grass. The administration of the different amounts of concentrate in the diet, as well as the slaughter weight were parameters that did not affect the IMF in Barbarine lambs [40]. Similarly, in another study comparing lambs feeding with pasture and those that are stall-fed also found no significant differences on IMF between the groups [39]. Other authors reported that the weaned treatment (early, middle and unweaned) did not influence the IMF [41]. In contrast, in our case, the extensively-reared lambs (unweaned) presented higher IMF than the intensively-reared lambs (weaned at 3 months age). This fact could partially explain the differences of IMF between groups, since a previous meta-analysis study demonstrated that lambs that received milk had higher IMF than those that only had access to the pasture alone [42]. In Addition, the weaning also affected the carcass weight, since the unweaned lambs had heavier carcasses (both under concentrate and pasture feeding regimes) than the weaned animals [43]. This result agrees perfectly with our findings, since animals reared in the extensive systems (unweaned) presented both higher IMF and higher carcass weight than lambs reared in the intensive production system. Moreover, despite the fact that the carcass weight was significantly higher in extensively-reared animals, the live weight at slaughter did not show significant differences between both treatments (13.54 vs. 12.44 kg for intensively-reared and extensively-reared lambs, respectively). Similar results were observed in the research of Boughalmi and Araba [44], who found that the feeding management system (grazing vs. grazing with supplement vs. concentrate diet) did not affected the live weight of Timahdite lambs. In another research and in accordance with our results, the authors observed that the grass-fed lambs presented higher values of IMF (2.4% vs. 1.4%) than lambs offered the concentrate diet [45]. In this case, the authors attributed this fact to the adaptation period after weaning to the indoor condition and the change of diet type, which could also explain the results found by us in the present study.

Nevertheless, the large differences found in the literature may be due to the distinct conditions of the studies (age and weight of slaughter, the diet composition, management, breed, gender, etc.). Some authors reported, in the same study, contrary behavior of IMF content between two breeds feeding with three systems [46]. In this case, the authors reported that Akkaraman lambs feeding with concentrate presented lower values of IMF than those that received pasture, while in the Anatolian Merino lambs the concentrate-feeding lambs presented the highest IMF content [46]. This demonstrated that multiple factors could affect this parameter. In fact, in a recent study, the authors reported that IMF is strongly affected by diet, sex and age [47]. Thus, it is difficult to attribute the differences in IMF values to a single factor. However, in the present study, the IMF differences could be attributable to the different rearing season of the animal groups (availability and quality of pasture), the weaning treatment and also due to the adaptation period of lambs to concentrate diet.

### 3.2. Volatile Profile

In this research, a total of 205 volatile compounds from *longissimus thoracis et lumborum* of the BEDM breed were identified in the headspace of raw meat employing the SPME/GC-MS technique. The compounds obtained were divided into nine families according to their chemical nature: hydrocarbons (linear, branched, aromatic and benzene-derived hydrocarbons), acids, alcohols, aldehydes, ketones, esters, ethers, furans and sulfur compounds.

#### 3.2.1. Hydrocarbons: Linear, Branched, Cyclic and Benzene-Derived

Table 2 displays the influence of the production system on the different hydrocarbons of the raw lamb meat. A total of 99 compounds belonging to this group were found, 70 in intensive-reared lambs and 48 in extensive-reared lambs. Concretely, in intensively-reared animals the hydrocarbons were distributed as follows: 9 linear hydrocarbons, 41 branched hydrocarbons, 16 cyclic hydrocarbons and 4 benzene-derived hydrocarbons. On the other hand, in extensively-reared lambs the hydrocarbons consisted of 11 linear hydrocarbons, 24 branched hydrocarbons, 11 cyclic hydrocarbons and 2 benzene-derived hydrocarbons.

As can be observed, the production system did not significantly affect the total hydrocarbon content, although this was slightly higher in lambs produced under extensive conditions (441.44 vs. 386.00 AU × 10^4^/g fresh meat). However, the total value of families of linear, cyclic and benzene-derived hydrocarbons were significantly (*p* < 0.001) affected by the production system. Specifically, lambs reared in the extensive system presented higher amounts of total linear hydrocarbons (130.93 vs. 26.15 AU × 10^4^/g fresh meat). On the contrary, lambs reared intensively had significantly (*p* < 0.001) higher concentrations of total cyclic hydrocarbons (36.81 and 29.89 AU × 10^4^/g fresh meat for intensive and extensive systems, respectively) and benzene-derived hydrocarbons (7.50 AU × 10^4^/g fresh meat for intensive and 2.12 AU × 10^4^/g fresh meat for extensive and 2.12). Numerically but not significantly, the branched hydrocarbons from intensively-reared animals were also higher (315.54 and 278.51 AU × 10^4^/g fresh meat for intensive and extensive production systems, respectively).

Most individual hydrocarbons were significantly (*p* < 0.05) affected by the production system apart from pentane, heptane, 1-undecene and heptylcyclohexane. Differences in volatile compounds attributed to the production system may arise from the origin of the animal feed, since some of these volatile compounds, such alkanes of more than 10 carbons, can be stored in fatty tissues through diet [48,49]. However, the individual trends varied depending on the substance in question. Thus, in intensively-reared lambs, the linear hydrocarbon that was found in the highest concentration was pentane (9.52 AU × 10^4^/g fresh meat), while for extensively-reared lambs it was decane (99.48 AU × 10^4^/g fresh meat). In the case of branched hydrocarbons, the highlights were 2,2,4,4-tetramethyloctane, with a concentration of 146.91 AU × 10^4^/g fresh meat, and heptadecane, with a concentration of 83.25 AU × 10^4^/g fresh meat, for intensive-raised and extensive-raised lambs, respectively. Moreover, for both production systems, the cyclic hydrocarbon with the highest presence was the same (namely bicyclo[3.2.0]hepta-2,6-diene) and shoed concentrations of 15.94 and 12.09 10 AU × 10^4^/g fresh meat for lambs reared in intensive and extensive systems, respectively. Within this group of hydrocarbons, it is also worth highlighting the presence of the terpene D-limonene in grass-fed lambs (0.90 AU × 10^4^/g fresh meat) and its absence in lambs fed with concentrate.

Additionally, it should be noted that benzene-derived hydrocarbons did show the same trend since all the compounds belonging to this group (namely ethylbenzene; benzene, 1,3-dimethyl-; benzene, n-butyl-; benzene, (1,1-dimethylethoxy)-) were found in significantly (*p* < 0.01) higher concentrations in lambs fed under the intensive production system. These results are in disagreement with those obtained by various authors who reported that benzene-derived hydrocarbons were produced to a greater extent in lambs fed by grazing than by concentrate [6,17,50]. This discrepancy is difficult to explain since normally benzene-derived hydrocarbons are related to the consumption of grass and, more specifically, to the carotenoids present in green plants [51] or even with the contaminants retained by these vegetables [52,53].

**Figure 1:**
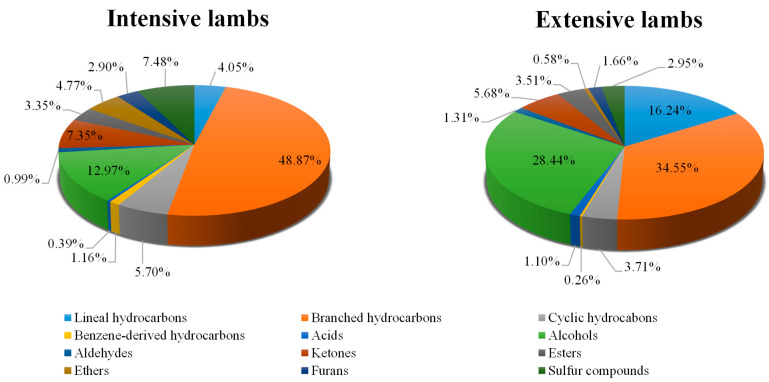
Volatile families of BEDM lamb *longissimus thoracis et lumborum* muscle (expressed as percentages) affected by the production system.

On the other hand, hydrocarbons constituted the largest family of volatile compounds detected in intensive and extensive systems (59.78% and 54.76%, respectively), with branched hydrocarbons being the volatile compounds most abundant in both diets (48.87% for lambs reared in intensive production system and 34.55% for animals reared in extensive production system) and benzene-derivatives being the least abundant ones (1.16 and 0.26% for intensively-reared and extensively-reared lambs, respectively) (Figure 1). In spite of the distributions of these percentages, linear hydrocarbons (which represent 4.05 and 16.24% in intensively-reared and extensively-reared lamb meat, respectively) and cyclic hydrocarbons (5.70% for intensively-reared and 3.71% for extensively-reared lambs) taken together with branched ones, in general, are not particularly important in contributing to the aroma of meat as they have high odor thresholds [29,54,55,56]. On the contrary, benzene-derived hydrocarbons, even those possessing a low percentage of the total volatile content, could have a significant contribution to the volatile pattern of lamb meat due to their low odor threshold [48,56,57].

#### 3.2.2. Acids

Seven acids were identified in the meat of BEDM lambs, four in samples from the intensive production system and six from the extensive system. Moreover, the production system significantly (*p* < 0.05) affected both the total amount of acids and that of each individual compound (Table 3). Specifically, extensively-reared lambs showed a higher concentration of all the acids determined with the exception of hexanoic acid, which appeared in intensively-reared lambs (1.33 AU × 10^4^/g fresh meat) while it was not detected in extensive farming lambs. In addition, the total amount of acids was also significantly (*p* < 0.001) higher in the lambs reared extensively (8.87 vs. 2.51 AU × 10^4^/g fresh meat).

Within this group, branched chain fatty acids, such as 4-methyloctanoic, 4-ethyloctanoic and 4-methylnonanoic acids, are of special interest because they are related to the specific aroma of lamb meat, contributing to the mutton-like aroma [6,58,59]. However, none of these compounds were detected in the lambs analyzed regardless of the production system employed.

The contribution of acids on the total volatile compounds was very low. Indeed, this family has been the least abundant in intensively-reared lambs and the second with the least presence in extensively-reared lambs. More concretely, total acids represented 0.39 and 1.10% of the total volatile substances in lambs reared in intensive and extensive production systems, respectively (Figure 1). This weak presence may be due to the fact that some acids, such as branched chain, are found mainly in adipose tissue since they are diminished in muscle tissue [58]. Furthermore, since branched fatty acids tend to increase with the age of the animals and are associated with older lambs of over two years [60], their presence in our study was limited due to the young age of the lambs (~4 months).

#### 3.2.3. Alcohols

In this study, 35 different alcohols (24 in animals from the intensive production system and 25 in animals from the extensive production system) were detected in the BEDM lambs meat (Table 3). As can be observed, all these compounds were significantly (*p* < 0.05) affected by the production system except for 1-heptanol, 1-octen-3-ol, 4-ethylcyclohexanol, 1-octanol and 2-octen-1-ol, (E)-, although in different manners. Nevertheless, it can be generally observed that the extensive production system tends to provide BEDM lamb meat with a higher presence of alcohols, since 20 of the compounds identified in this group were found in significantly (*p* < 0.05) higher concentrations compared to their intensively-reared counterparts. Moreover, BEDM lambs reared in the extensive production system showed a total content of alcohols significantly (*p* < 0.001) higher than those reared in the intensive system (229.30 and 83.76 AU × 10^4^/g fresh meat, respectively). This occurrence could be due to the fact that some alcohols, such as 1-pentanol and 1-hexanol, are related to the degradation of their homologous aldehydes during lipid oxidation [61,62]. In this regard, a previous study demonstrated that the BEDM lambs reared extensively have very high contents of polyunsaturated fatty acids (specially n-3 PUFA) [9], which are more susceptible to oxidation [63] and can explain the results observed on the lipid-derived volatile compounds behavior. Thus, in our study, 1-pentanol alone has been identified in extensively-reared lambs (33.61 AU × 10^4^/g fresh meat) and 1-heptanol has shown a concentration of 7.84 AU × 10^4^/g fresh meat in extensively-reared lambs compared to 3.70 AU × 10^4^/g in intensively-reared lambs. Nevertheless, the greater levels of 1-hexanol in extensively-reared lambs contrasts with the fact that this alcohol comes from the autoxidation of linoleic acid [63,64], which is typically present in concentrates made from grains [65]. Despite these observations, other studies have also found that grass-raised ewes showed higher amounts of 1-hexanol in meat than intensively-reared ewes [18]. In addition, 1-pentanol and 1-hexanol could positively affect the aroma of lamb meat since 1-pentanol is characterized by its pleasant, sweet or fruity odor, while 1-hexanol has a herbal and fatty odor [29,48,66]. Furthermore, the lambs reared in the extensive production system displayed a concentration of 1-butanol that is significantly higher than that of the lambs reared intensively (29.94 vs. 2.01 AU × 10^4^/g fresh meat). These results agree with the fact that meat from BDEM lambs reared in extensive system presented high amounts of linoleic acid [9], which is the main precursor of this volatile compound (derived from oxidation reactions) [63].

On the other hand, two alcohols (namely benzyl alcohol and 2,4-di-tert-butylphenol) have been identified, which could be related to the diet based on grass as they are phenolic compounds [19]. However, in the meat of BEDM lambs reared in extensive production system (fed with grass) none of these two compounds were identified, while in those fed with concentrate (intensive production system) values of 0.27 and 2.52 AU × 10^4^/g fresh meat were obtained for benzyl alcohol and 2,4-di-tert-butylphenol, respectively. These results are similar to those indicated by other authors, which suggest that not all phenolic compounds are related to grass [14,67].

Regarding the 1-octanol, this alcohol was not significantly affected (*p* > 0.05) by breeding, obtaining very similar values for both types of lambs (3.25 and 3.95 AU × 10^4^/g fresh meat for intensively-reared and extensively-reared lambs, respectively). Similarly, 1-octen-3-ol has not been significantly (*p* > 0.05) affected by the production system, since both lambs showed concentrations in the same range (39.65 and 33.1 AU × 10^4^/g for intensively- and extensively-reared lambs, respectively). This could be due to the fact that 1-octen-3-ol is a compound that arises from several pathways [48]; it is a volatile substance derived from lipid oxidation that is frequently reported in meat and meat products [63,68]. These facts are in agreement with those obtained by Sivadier et al. [17] who observed that 1-octen-3-ol content did not depend on the diet supplied. In addition, although they are normally of lower molecular weight, there are various alcohols that are considered to be of metabolic origin; thus, they are not affected by the diet provided [69].

With respect to the contribution of alcohols over the total volatile content, this family is the second most abundant in both production systems. Specifically, this group represents 12.97 and 28.44% in the lambs reared intensively and extensively, respectively (Figure 1). Despite this, alcohols have a debatable high odor threshold and their contribution to volatile flavor is less than that of other compounds such as aldehydes [70]. However, various alcohols, such as 1-pentanol, may contribute to the lamb aroma on account of their low odor threshold and their mild, fruit and balsamic aroma [66,71].

#### 3.2.4. Aldehydes

In our work, the lambs reared extensively showed a significantly (*p* < 0.001) higher total aldehyde value than lambs reared intensively (10.53 vs. 6.40 AU × 10^4^/g fresh meat), which is inconsistent with what was obtained in Almela et al. [5]. Within the aldehyde family, 14 compounds were identified (11 in lambs from intensive production and 8 in lambs from extensive production system) (Table 4), among which hexanal was the only volatile compound that was not significantly (*p* > 0.05) affected by the production system and similar concentrations in both groups of lambs were found (1.07 and 0.97 AU × 10^4^/g fresh meat). This suggests that the meat from both productions systems could show similar lipid oxidation states, since hexanal is assumed to be one of the main indicators of lipid oxidation [72,73]. However, other aliphatic saturated aldehydes found in our research, apart from hexanal, are also considered as indicators of lipid oxidation in raw meat because they are derived from the degradation of hydroperoxides [63,74]. This is the case of the octanal and nonanal aldehydes, which are derived from the oxidation of oleic acid [63,75]. Concretely, octanal was the one that was found in greater presence in the lambs reared under intensive conditions (2.30 AU × 10^4^/g fresh meat), while it did not appear in the lambs reared extensively. This fact could indicate that intensively-reared lambs may have a greater intensity of rancid odor, since previous studies have found that octanal is the aldehyde that presented the highest correlation with this parameter in lamb meat packed under a protective atmosphere [76]. On the contrary, nonanal was only detected in extensively-reared lambs since it is the aldehyde that appears in the highest concentration in the meat of these animals (2.56 AU × 10^4^/g fresh meat), after 2-propenal with a concentration of 4.04 10^4^/g fresh meat, which would provide a plastic and soapy aroma [77].

Another important aldehyde is heptanal, which is usually an indicator of animal diets rich in linoleic acid, since it is an aldehyde that appears after the oxidation of this fatty acid [64]. In this manner, it would be expected that the lambs reared in the intensive system would obtain higher concentrations of heptanal than those reared extensively because linoleic acid is typically present at high quantities in cereal grains [65]. Conversely, in our research, lambs from extensive production system displayed significantly (*p* < 0.001) higher amounts of heptanal than intensive-reared lambs (0.95 vs. 0.27 AU × 10^4^/g fresh meat). It is important to highlight that in a previous study, the BDEM lambs reared in extensive system also presented high amounts of this fatty acid, which explains our findings [9]. These results are consistent with those shown by Vasta et al. [18], who found that milk from grass-fed ewes had higher concentrations of heptanal than those fed a grain-based diet. Therefore, it is not easy to unambiguously link an aldehyde compound with a lamb feeding or production system [14].

On the other hand, the fraction corresponding to the group of aldehydes with respect to the total volatiles was very low in both groups (0.99 and 1.31%, for intensive-reared and extensive-reared lambs, respectively) (Figure 1). Specifically, it is the second and third group of volatile compounds that are the less abundant of the nine divisions in lambs produced in intensive and extensive systems, respectively. This fact is in disagreement with the results reported by other authors who found that the aldehyde family generally represents the main contributors to the volatile fraction extracted from ruminant meat [18,78]. Despite this discrepancy, aldehydes remain one of the most important volatile compounds because they are the main indicators of rancidity in meat due to their low odor threshold [79,80].

#### 3.2.5. Ketones

A total of 24 ketones were identified in the BEDM lamb meat (17 in intensively-reared and 18 in extensively-reared lambs). As shown in Table 4, the production system did not significantly (*p* > 0.05) affect the total amount of ketones, although it was slightly higher in extensively-reared lambs (47.48 vs. 45.81 AU × 10^4^/g fresh meat). Despite the fact that the total content of this family was not affected by production system, each individual ketone showed significant (*p* < 0.05) differences according to the production system employed, with the exception of pyrolo[3,2-d]pyrimidin-2,4(1H,3H)-dione; 3-nonanone and 2(3H)-furanone, 5-ethyldihydro-, also known as γ-hexalactone, which could be related to the metabolism of the ruminants since certain ketones are considered to be of metabolic origin [69]. Conversely, there are ketones that are derived from the diet [18]. This is the case of 2,3-octanedione, which has been considered by several studies as a typical compound present in grass-fed animals meat [18,20]. However, the results obtained by Resconi et al. [81] and Gravador et al. [68] did not identify 2,3-octanedione in lambs regardless of their diet.

On the other hand, 2,3-butanedione (diacetyl), was linked with grain diets [23]. This event is in agreement with the results obtained in our work, since it has been observed that only lambs reared in the intensive system had 2,3-butanedione (15.54 AU × 10^4^/g fresh meat), while this diketone was not identified in extensively-reared animals. In fact, 2,3-butanedione also stands out for being the ketone that appears in greater abundance in lambs fed with concentrate. Additionally, the presence of 2-heptanone and 2-butanone are associated with grain-based diets [16,65]. Despite this, in our study, it was found that lambs reared extensively presented significantly (*p* < 0.001) higher amounts of 2-heptanone, 2-octanone and 2-butanone (8.67 vs. 1.68 AU × 10^4^/g fresh meat for 2-heptanone; 4.08 vs. 2.16 AU × 10^4^/g fresh meat for 2-octanone; and 1.93 vs. 0.72 AU × 10^4^/g fresh meat for 2-butanone) and even 2-heptanone, which is the ketone that was detected in greater abundance in these lambs. These unexpected outcomes are consistent with those obtained by Vasta et al. [18] who did not observe significant differences in this 2-ketones, yet did find slightly higher amounts in lambs fed with grass than with concentrate. Additionally, the high proportion of these ketones in animals reared in the extensive production system could be due to 2-ketones being derived from lipid oxidation [63] and BEDM lambs that are extensively-reared had the highest amounts of PUFA [9], which promotes their formation. Contrary, 2-nonanone was identified in a significantly higher concentration (*p* < 0.01) in intensively-reared lambs (0.86 and 0.65 AU × 10^4^/g fresh meat for intensively-reared and extensively-reared animals, respectively). Although the value of 2-nonane is significant higher in lambs from intensive systems than in lambs from extensive production system, it is important to mention that the difference of content between both groups of animals was less than those described for the aforementioned 2-ketones. This ketone (2-nonanone) possesses a “fatty, oily, fruity” odor and has previously been associated with a lamb flavor [82,83], which could indicate that lambs reared in intensive production system could show a stronger flavor linked to this compound.

Furthermore, it should be noted that up to four different lactones were identified in the lamb meat, namely butyrolactone; 2(3H)-furanone, dihydro-5-methyl-; 2(3H)-furanone, 5-ethyldihydro-; and 2(3H)-furanone, dihydro-5-pentyl. It has been previously pointed out that this type of lactones have been linked to grain-based diets [20,21] due to its higher content of oleic and linoleic acids compared to pasture [84]. This is because lactones arise from the corresponding hydroxy-fatty acids [85], which in turn are formed in the rumen by the oxidation of dietary oleic and linoleic acids [86]. However, in our work, only butyrolactone seemed to follow the trend expected, since it was found in intensively-reared lambs (2.71 AU × 10^4^/g fresh meat) and not in extensively-reared lambs. On the contrary, 2(3H)-furanone, dihydro-5-methyl- and 2(3H)-furanone, dihydro-5-pentyl- were only identified in lambs produced in extensive systems and obtained concentrations of 8.53 and 0.36 AU × 10^4^/g fresh meat in these lambs, respectively. Finally, 2(3H)-furanone, 5-ethyldihydro- was not significantly (*p* > 0.05) affected by the production system.

Regarding the contribution of ketones on the total volatile compounds, this family represented 7.35 and 5.68% of the total volatile substances in intensively-reared and extensively-reared lambs, respectively (Figure 1). This percentage was slightly lower than that reported by Krvavica et al. [87], who observed ketone values of around 9% in lamb of the Lika breed. Despite this, the percentage of ketones is relatively high, since this group is the fourth most abundant family for intensively-reared lambs and the third for extensively-reared lambs within the nine groups. This occurrence combined with the fact that ketones have a low perception threshold [56,82] renders this group a notable contributor to the meat flavor [73].

#### 3.2.6. Esters, Ethers, Furans and Sulfur Compounds

Sixteen different esters were detected in BEDM lambs meat (8 in intensively-reared and 11 in extensively-reared lambs), which were significantly (*p* < 0.001) affected by the production system except for a single compound, namely 2-butenoic acid, 2-methyl-, 2-methylpropyl ester, which did not suffer significant (*p* > 0.05) variations (Table 5). In general, esters were found to a greater extent in lambs reared in extensive systems since 10 of the 16 compounds obtained significantly (*p* < 0.001) higher concentrations in these animals. In addition, the total content of esters was also significantly (*p* < 0.05) higher in the lambs reared in extensive systems compared to those reared in intensive systems (28.30 vs. 21.62 AU × 10^4^/g fresh meat). These differences could be related to the possible variability of the fatty acid profile of lambs [88] because the main origin of esters is the esterification of carboxylic acids [89]. Despite the differences, previous studies have shown that the contribution of esters to the aromatic profile of lamb meat may be low [68]; several authors did not even detect these compounds [17,81,90,91] or detected a low number of esters [37,76,87,88]. Therefore, although the fraction of esters to the total volatile compounds was relatively high (3.35 in intensively-reared lambs and 3.51% for lambs reared under extensive conditions) (Figure 1), their presence may not contribute to the overall aroma of the lamb meat.

Regarding the ethers group, only three different compounds were identified (Table 5). Two were found in intensively-reared lambs (namely, ether, 2-ethylhexyl tert-butyl and decyl heptyl ether) and one in lambs raised extensively (namely, ether, 3-butenyl pentyl). All these individual compounds as well as their total content were significantly (*p* < 0.001) affected by the production system. Specifically, the lambs fed under the intensive diet showed significantly (*p* < 0.001) higher amounts of this group (30.83 vs. 4.69 AU × 10^4^/g fresh meat). In addition, ethers represented 4.77% of the total volatile content in lambs from the intensive system and occupies the fifth position of the nine families, while this group only accounted for 0.58% of the total volatile content in lambs from the extensive system and is the family that appears in the lowest presence (Figure 1). The literature consulted did not frequently find these compounds in lamb meat and, in some cases, were non-existent in many investigations [18,34,91,92]. Furthermore, it was observed that ethers were not relevant compounds in the aroma of lambs [93] and some of these substances could be found in lamb due to their possible use as insecticides, acaricides and fumigants for the soil [48].

On the other hand, four furans were identified in both lambs (Table 5), except for furan, 2,3-dihydro-, which was only found in lambs reared in extensive systems at a concentration of 1.76 AU × 10^4^/g fresh meat. Specifically, the furan that appeared in the highest concentration was furan, 2-pentyl in both production systems, which has been frequently identified in lamb meat [76,83,87,92] and related with lipid oxidation [29,75,83], green bean and butter flavors [66]. According to Fruet et al. [94], feeding with grass provided animals with significantly (*p* < 0.001) lower concentrations of furan, 2-pentyl (6.41 AU × 10^4^/g fresh meat compared to the 17.49 AU × 10^4^/g fresh meat of lambs reared intensively). This fact could reveal that the grass-based diet has a higher content of α-tocopherol, since the formation of furan, 2-pentyl is negatively correlated with said antioxidant [24]. On the contrary, the rest of furans were found in a higher concentration in lambs reared in the extensive range, being significant (*p* < 0.001) in the case of furan, 2-ethyl- and furan, 2,3-dihydro-. Despite this, the total content of furans remained significantly (*p* < 0.01) higher in intensively-reared lambs (18.13 vs. 13.38 AU × 10^4^/g fresh) due to their higher contribution of furan, 2-pentyl. Additionally, the furan group represented a percentage of 2.90 and 1.66% of the total volatile compounds found in intensive and extensive lambs, respectively (Figure 1). These fractions are not very high, since furans represent the sixth and seventh family in lambs reared extensively and intensively, respectively. However, their occurrence can be very important, since these compounds are potential contributors to the rancid aroma of meat [76].

Finally, in the present research three sulfur compounds were identified in both intensively-reared and extensively-reared lambs, which were significantly (*p* < 0.01) affected by the production system (Table 5). Specifically, intensively-reared lambs produced a significantly (*p* < 0.01) higher concentration for the total content of these substances (48.30 vs. 23.81 AU × 10^4^/g fresh). In disagreement with these findings, several studies displayed that sulfur compounds were present at higher concentration in grass-feed animals compared to animals fed with concentrates [6,14]. However, the higher content in our research can be related to the amount of the carbon disulfide, since it turned out to be the only sulfurous compound found in high levels in lambs reared intensively (45.75 vs. 21.34 AU × 10^4^/g fresh). Despite this difference, disulfide carbon was the most abundant sulfur compound detected in both production systems. This substance can be derived from the enzymatic proteolysis of sulfur-containing amino acids [95] and/or from dithio-carbamate fungicides employed in agriculture [96]. Disulfide carbon could be important in the aromatic profile of lamb as it has been found to contribute to the overall aroma of packed meat [95] and possess a pleasant, sweet or ether-like odor [48]. Furthermore, Karabagias [48] concluded that carbon disulfide could be considered as a typical volatile compound of raw lamb meat. Contrary, dimethyl sulfide and dimethyl sulfone have been detected in significantly (*p <* 0.01) higher amounts in extensively-reared animals. These compounds are important because they can create adverse flavors in extensively-reared lambs. In this respect, dimethyl sulfone has been associated with unfavorable sensory descriptors [6]. Regarding the contribution of sulfur compounds on the volatile profile, this family represented 7.48 and 2.95% of the total volatile compounds in the lambs reared intensively and extensively, respectively. This presence can be considered important since, in addition to being the third and fifth most abundant family in intensively-fed and extensively-fed lambs, sulfur compounds contribute to the general aroma of meat [95].

A further consideration on the overall meat aroma profile is that lambs fed extensively displayed a significantly (*p* < 0.001) higher concentration of total volatile compounds (806.13 vs. 645.13 AU × 10^4^/g fresh meat). This difference could suggest that extensively-reared lambs may have a higher flavor intensity than intensively-reared lambs. Furthermore, these outcomes are in line with those encountered by other studies, which found a greater flavor intensity in the meat of animals fed with pasture [15,18] or whose mothers were grazing at a pasture [18,97] in comparison to meat from animals fed with concentrates. This occurrence could be due to the fact that extensively-reared lambs contain a significantly (*p* < 0.001) higher fat content than intensively-reared lambs (Table 1), which can generate a greater amount of volatile compounds. In our study no significant (*p >* 0.05) correlations were found between intramuscular fat and total volatile content for either of the two production systems analyzed (r = 0.030; *p >* 0.05, for intensively-reared lambs; and r = 0.127; *p >* 0.05, for extensively-reared lambs). Similarly, the correlations between intramuscular fat and the different families of volatile compounds were low (r < 0.450) and not significant (*p >* 0.05) in any case (except for the correlation found in extensively-reared lambs for total ethers, where a significant correlation was observed (r = 0.634; *p* < 0.05)). However, when the individual volatile compounds were analyzed, a significant correlation (both positive and negative, depending on the volatile substance) between IMF and 180 compounds was detected. Among these compounds, it is important to highlight that strong, significant and positive correlations (r > 0.6 and *p* < 0.01) between IMF and the most important lipid-derived compounds, such as 1-propanol (r = 0.708; *p* < 0.01), 1-pentanol (r = 0.642; *p* < 0.01), 1-hexanol (r = 0.633; *p* < 0.01), nonanal (r = 0.639; *p* < 0.01), 2-butanone (r = 0.654; *p* < 0.01), 2-pentanone (r = 0.645; *p* < 0.01) and 2-heptanone (r = 0.684; *p* < 0.01) was observed. This fact confirms that the influence of the production system on the lipid content and that the lipid composition could likely be one of the main important factors for the release of characteristic volatile compounds that mainly consists of lipid-derived compounds, which, generally speaking, have a high impact on meat aroma due to their low odor thresholds.

## 4. Conclusions

The use of two different production systems (intensive and extensive) significantly affected the proximate composition of the BEDM lamb meat. Animals reared in an extensive production system presented the highest values of IMF and protein, while it demonstrated the lowest values for moisture and ash. In the same manner, the total concentration of volatile compounds was also affected, being higher in lambs reared in extensive regime, although it did not seem related to intramuscular fat content. However, the intramuscular fat content had a strong effect on the most individual volatile content derived from lipid reactions (lipolysis, lipid oxidation, etc.). Furthermore, most of the individual volatile compounds were also influenced by the production system, which could be related to both, and specific compounds linked to the diet or the variation of lipid fraction (lipid content and fatty acids), which highly influenced the release of lipid-derived volatile compounds.

## Figures and Tables

**Table 1:** Effects of the production system on the proximate composition of BEDM lamb *longissimus thoracis et lumborum* muscle.

	Intensive	Extensive	SEM	Sig.
Moisture (%)	78.00	75.91	0.290	***
Intramuscular fat (%)	0.49	1.51	0.132	***
Protein (%)	19.32	20.92	0.234	***
Ash (%)	1.37	1.20	0.022	***

SEM: Standard error of the mean. Sig.: Significance. *** (*p* < 0.001).

**Table 2:** Effects of the production system on hydrocarbons (expressed as AU × 10^4^/g fresh weight) of BEDM lamb *longissimus thoracis et lumborum* muscle.

	LRI	*m*/*z*	Intensive	Extensive	SEM	Sig.
**Linear hydrocarbons**						
Butane	496	43	0.00	3.68	0.480	***
Pentane	500	43	9.52	11.75	0.961	ns
Heptane	700	71	1.00	1.29	0.100	ns
Octane	800	85	0.00	8.49	0.884	***
4-Octene, (E)-	841	55	0.00	2.42	0.241	***
Decane	1000	57	3.45	99.48	10.190	***
Undecane	1100	57	5.53	0.27	0.633	***
1-Undecene	1129	83	0.68	0.48	0.057	ns
Dodecane	1200	57	3.33	1.30	0.263	***
Hexadecane	1210	57	0.00	1.30	0.139	***
1-Tetradecene	1260	71	0.23	0.00	0.030	***
Tridecane	1300	57	1.55	0.47	0.146	***
Tetradecane	1400	57	0.87	0.00	0.101	***
**Total linear hydrocarbons**			26.15	130.93	11.279	***
**Branched hydrocarbons**						
Pentane, 2-methyl-	541	71	0.51	2.45	0.199	***
Pentane, 3-methyl-	550	56	1.18	29.79	2.839	***
Butane, 2,2,3,3-tetramethyl-	656	57	0.00	10.92	1.208	***
Hexane, 2,2-dimethyl-	656	57	17.00	0.00	2.111	***
Pentane, 2,3-dimethyl-	675	56	0.66	0.00	0.091	***
Pentane, 2,3,4-trimethyl-	759	71	21.00	0.09	2.375	***
Pentane, 2,3,3-trimethyl-	767	70	46.63	0.19	5.061	***
Pentane, 3-ethyl-	774	70	0.00	0.48	0.060	***
Hexane, 2,3-dimethyl-	774	70	1.45	0.00	0.214	***
1-Pentene, 3-ethyl-2-methyl-	778	55	1.37	0.00	0.169	***
3,4-Dimethyl-2-hexene	778	83	1.38	0.00	0.193	***
1-Pentene, 4,4-dimethyl-	788	57	0.00	0.61	0.084	***
Butane, 2,2,3-trimethyl-	789	85	0.56	0.00	0.067	***
Hexane, 2,2,5-trimethyl-	806	57	18.43	0.37	2.071	***
Heptane, 3-methylene-	820	70	5.10	0.00	0.692	***
Heptane, 3,4,5-trimethyl-	850	85	0.00	8.92	0.894	***
Pentane, 2,3,3,4-tetramethyl-	850	84	0.00	1.63	0.191	***
Heptane, 2,3-dimethyl-	850	85	1.42	0.00	0.180	***
Heptane, 2,6-dimethyl-	863	88	0.37	0.00	0.043	***
Heptane, 3-ethyl-	917	57	1.50	0.00	0.166	***
Nonane, 3,7-dimethyl-	925	57	1.08	0.00	0.129	***
Heptane, 2,2,4-trimethyl-	933	57	1.99	1.20	0.168	*
Heptane, 3,3,5-trimethyl-	947	71	0.00	0.46	0.048	***
Octane, 3,3-dimethyl-	947	71	1.95	0.00	0.218	***
Hexane, 2,3,4-trimethyl-	948	57	0.00	0.43	0.046	***
Pentane, 2,2-dimethyl-	948	57	1.29	0.00	0.141	***
3-Ethyl-2-methyl-1-heptene	996	84	0.89	0.00	0.100	***
Heptane, 3-ethyl-5-methylene-	998	70	0.00	1.94	0.226	***
2,3-Dimethyl-1-hexene	1037	55	1.80	0.00	0.191	***
Pentane, 3,3-dimethyl-	1046	71	3.01	0.00	0.349	***
1-Hexene, 3-methyl-	1062	70	3.04	0.00	0.386	***
(Z)-4-Methyl-2-hexene	1072	98	0.89	0.00	0.096	***
2,2,4,4-Tetramethyloctane	1078	57	146.91	17.31	16.485	***
1-Hexene, 5,5-dimethyl-	1090	57	0.00	58.12	6.290	***
Nonane, 5-butyl-	1097	127	1.45	0.00	0.172	***
Nonane, 5-(2-methylpropyl)-	1097	71	7.01	0.00	0.868	***
Heptane, 2,3,4-trimethyl-	1097	57	0.00	57.47	6.317	***
Dodecane, 2,6,10-trimethyl-	1097	57	14.27	0.00	1.805	***
Heptane, 2,2-dimethyl-	1101	57	0.93	0.00	0.141	***
Decane, 6-ethyl-2-methyl-	1104	57	0.00	83.25	8.323	***
Heptane, 3,3,4-trimethyl-	1135	71	0.00	0.50	0.060	***
Nonane, 2-methyl-	1136	57	0.69	0.00	0.084	***
Hexane, 1-(hexyloxy)-3-methyl-	1147	57	2.34	0.00	0.275	***
2-Undecene, 9-methyl-, (Z)-	1152	98	2.66	0.00	0.286	***
4-Undecene, 5-methyl-	1165	168	0.30	0.00	0.036	***
Pentane, 3,3-diethyl-	1181	98	0.34	0.00	0.036	***
2-Undecene, 3-methyl-, (Z)-	1203	70	0.60	0.00	0.065	***
Octane, 2,4,6-trimethyl-	1210	71	0.00	0.86	0.089	***
5-Ethyl-1-nonene	1224	83	0.32	0.00	0.039	***
1-Decene, 2,4-dimethyl-	1224	70	0.42	0.00	0.052	***
Hexane, 2-methyl-4-methylene-	1227	71	0.00	0.42	0.046	***
Heptadecane, 8-methyl-	1227	71	1.12	0.00	0.142	***
Undecane, 5-ethyl-	1242	57	1.09	0.00	0.157	***
Dodecane, 2-methyl-	1257	57	0.22	0.00	0.029	***
1-Undecene, 8-methyl-	1260	97	0.34	0.00	0.046	***
Tridecane, 3-methyl-	1331	57	0.00	0.39	0.039	***
Heptane, 2,4-dimethyl-	1349	71	0.00	0.35	0.036	***
5,5-Dibutylnonane	1358	71	0.00	0.36	0.037	***
**Total branched hydrocarbons**			315.54	278.51	15.461	ns
**Cyclic hydrocarbons**						
Cyclopentane, 1,2-dimethyl-, cis-	666	56	0.49	3.39	0.336	***
Cyclohexane, methyl-	720	83	0.00	5.77	0.628	***
Bicyclo[3.2.0]hepta-2,6-diene	810	91	15.94	12.09	0.788	*
Cyclopentane, 1,2,3-trimethyl-	820	56	0.62	0.00	0.083	***
Cyclooctane	822	70	0.00	2.75	0.275	***
Cyclohexane, 1,3-dimethyl-, cis-	840	97	2.02	0.00	0.228	***
Cyclohexane, 1,3-dimethyl-	840	97	0.49	0.00	0.059	***
Cyclobutane, 1,1,2,3,3-pentamethyl-	938	70	1.69	0.00	0.173	***
Cyclopropane, 1-methyl-2-pentyl-	942	55	0.34	0.00	0.037	***
Bicyclo[3.1.1]hept-2-ene, 3,6,6-trimethyl-	992	93	2.74	0.00	0.289	***
Cyclopentane, 1,2,3,4,5-pentamethyl-	996	69	0.86	0.00	0.094	***
Cyclohexane, butylidene-	1042	67	0.00	0.72	0.078	***
Cyclodecene, (Z)-	1042	67	3.64	0.00	0.371	***
Cyclopropane	1063	41	3.04	0.00	0.318	***
Cyclohexane, 1,2-diethyl-1-methyl-	1075	125	0.52	0.00	0.057	***
Cyclopentane, pentyl-	1084	68	1.87	0.00	0.193	***
D-Limonene	1085	93	0.00	0.90	0.099	***
Cyclooctane, methyl-	1129	55	0.00	0.74	0.115	***
Cyclopentane, 1-ethyl-1-methyl-	1143	83	0.00	2.05	0.216	***
Butane, 2-cyclopropyl-	1165	70	0.98	0.00	0.116	***
Cyclododecane	1249	83	0.62	0.00	0.072	***
Heptylcyclohexane	1322	82	0.95	0.78	0.094	ns
Cyclopropane, 1,1,2,3-tetramethyl-	1374	71	0.00	0.46	0.051	***
Cyclohexane, octyl-	1444	82	0.00	0.24	0.024	***
**Total cyclic hydrocarbons**			36.81	29.89	0.980	***
**Benzene-derived hydrocarbons**						
Ethylbenzene	928	91	0.82	0.00	0.087	***
Benzene, 1,3-dimethyl-	937	106	2.54	1.58	0.177	**
Benzene, n-butyl-	1118	91	0.91	0.00	0.097	***
Benzene, (1,1-dimethylethoxy)-	1137	94	3.24	0.54	0.286	***
**Total benzene-derived hydrocarbons**			7.50	2.12	0.590	***
**TOTAL HYDROCARBONS**			386.00	441.44	18.278	ns

SEM: Standard error of the mean. Sig.: Significance. * (*p* < 0.05); ** (*p* < 0.01); *** (*p* < 0.001); ns: no significant difference.

**Table 3:** Effects of the production system on acids and alcohols (expressed as AU × 10^4^/g fresh weight) of BEDM lamb *longissimus thoracis et lumborum* muscle.

	LRI	*m*/*z*	Intensive	Extensive	SEM	Sig.
**Acids**						
Acetic acid	696	60	0.05	0.45	0.044	***
2-Propenoic acid	709	55	0.00	3.64	0.393	***
Butanoic acid	929	60	1.10	1.91	0.164	*
Pentanoic acid	1101	60	0.00	1.67	0.204	***
Hexanoic acid	1102	60	1.33	0.00	0.158	***
Pentanoic acid, 2-methyl-, anhydride	1157	99	0.04	0.95	0.106	***
Nonanoic acid	1314	60	0.00	0.26	0.031	***
**Total acids**			2.51	8.87	0.678	***
**Alcohols**						
Glycidol	499	44	2.10	90.02	12.230	***
1-Propanol	570	59	0.20	1.05	0.100	***
1-Butanol	709	56	2.01	29.94	3.053	***
1-Butanol, 3-methyl-	814	55	0.22	1.89	0.199	***
1-Butanol, 2-methyl-	818	57	0.00	4.70	0.506	***
1-Pentanol	855	55	0.00	33.61	3.542	***
Cyclobutanol, 2-ethyl-	875	56	1.03	0.00	0.130	***
2-Octen-1-ol, (Z)-	875	67	0.72	0.00	0.098	***
2,3-Butanediol, [S-(R*,R*)]-	929	45	3.44	0.00	0.408	***
DL-2,3-Butanediol	931	45	0.00	0.73	0.084	***
1-Butanol, 3-methyl-, acetate	952	55	0.05	1.37	0.306	*
1-Hexanol	967	55	3.70	7.84	0.632	***
1-Heptanol	1062	70	4.66	5.29	0.449	ns
1-Octen-3-ol	1068	57	39.65	33.11	3.525	ns
Ethanol, pentamethyl-	1079	59	0.00	0.72	0.074	***
2,3,4-Trimethyl-1-pentanol	1099	71	6.45	0.00	0.795	***
1-Hexanol, 2-ethyl-	1113	57	4.91	2.62	0.371	***
1-Hexanol, 5-methyl-2-(1-methylethyl)-	1128	71	0.94	0.00	0.109	***
1-Undecanol	1129	69	0.00	0.32	0.035	***
4-Ethylcyclohexanol	1130	81	0.24	0.41	0.052	ns
Benzyl alcohol	1145	108	0.27	0.00	0.030	***
5-Methyl-1-heptanol	1143	70	1.11	2.70	0.220	***
1-Octanol	1147	56	3.25	3.95	0.289	ns
2-Octen-1-ol, (E)-	1148	57	1.62	1.94	0.203	ns
3-Octen-2-ol, (E)-	1148	67	0.00	1.02	0.117	***
3-Octen-1-ol, (Z)-	1149	81	0.69	0.00	0.116	**
1-Butanol, 2-methyl-, trifluoroacetate	1152	70	3.35	0.00	0.361	***
1,8-Octanediol	1168	55	0.00	4.04	0.562	***
6-Undecanol	1183	55	0.00	0.78	0.093	***
4-Methyl-5-decanol	1184	83	0.42	0.00	0.066	***
1-Butanol, 3,3-dimethyl-	1189	56	0.00	0.37	0.038	***
1,9-Nonanediol	1224	55	0.00	0.20	0.021	***
1-Nonanol	1224	56	0.21	0.15	0.014	*
1-Butanol, 2-methyl-, propanoate	1349	57	0.00	0.52	0.053	***
2,4-Di-tert-butylphenol	1456	191	2.52	0.00	0.353	***
**Total alcohols**			83.76	229.30	16.597	***

SEM: Standard error of the mean. Sig.: Significance. * (*p* < 0.05); ** (*p* < 0.01); *** (*p* < 0.001); ns: no significant difference.

**Table 4:** Effects of the production system on aldehydes and ketones (expressed as AU × 10^4^/g fresh weight) of BEDM lamb *longissimus thoracis et lumborum* muscle.

	LRI	*m*/*z*	Intensive	Extensive	SEM	Sig.
**Aldehydes**						
Propanal, 2-methyl-	556	72	0.00	0.21	0.021	***
Butanal, 3-methyl-	659	58	0.26	0.57	0.050	***
Butanal, 2-methyl-	671	57	0.14	1.00	0.097	***
2-Butenal	841	70	0.39	0.00	0.047	***
Hexanal	874	56	1.07	0.97	0.109	ns
Heptanal	987	70	0.27	0.95	0.100	***
Hexanal, 3-methyl-	988	55	0.23	0.00	0.034	***
Hexanal, 3,3-dimethyl-	1006	69	0.92	0.00	0.099	***
Octanal	1084	84	2.30	0.00	0.276	***
Benzeneacetaldehyde	1139	91	0.09	0.23	0.026	**
2-Propenal	1148	55	0.00	4.04	0.437	***
Nonanal	1168	57	0.00	2.56	0.278	***
2-Decenal, (E)-	1298	83	0.40	0.00	0.039	***
2-Decenal, (Z)-	1299	70	0.32	0.00	0.040	***
**Total aldehydes**			6.40	10.53	0.555	***
**Ketones**						
2,3-Butanedione	589	86	15.54	0.00	1.711	***
2-Butanone	593	72	0.72	1.93	0.138	***
2-Pentanone	724	86	0.23	0.66	0.047	***
3-Pentanone	735	57	6.65	0.00	0.909	***
2,3-Pentanedione	739	100	0.00	0.79	0.097	***
1,5-Heptadien-4-one, 3,3,6-trimethyl-	779	83	0.00	0.99	0.115	***
Cyclobutanone, 2,2,3-trimethyl-	815	70	0.00	2.90	0.306	***
3-Heptanone	973	57	0.32	2.32	0.258	***
2-Heptanone	980	58	1.68	8.67	0.851	***
Pyrolo[3,2-d]pyrimidin-2,4(1H,3H)-dione	1057	151	9.83	8.21	0.456	ns
3-Ethylcyclopentanone	1058	83	0.00	0.37	0.042	***
4-Octanone, 5-hydroxy-2,7-dimethyl-	1059	69	0.00	2.49	0.283	***
Butyrolactone	1061	86	2.71	0.00	0.283	***
4-Hexen-3-one, 5-methyl-	1062	83	0.41	0.00	0.050	***
3-Heptanone, 5-methyl-	1069	99	3.28	0.00	0.395	***
5-Hepten-2-one, 6-methyl-	1073	68	0.74	0.49	0.059	*
2-Octanone	1077	58	2.16	4.08	0.334	*
2(3H)-Furanone, dihydro-5-methyl-	1095	56	0.00	8.53	1.108	***
5-Hexen-3-one	1151	98	0.90	1.24	0.076	*
3-Nonanone	1155	72	0.61	0.57	0.050	ns
2-Nonanone	1161	58	0.86	0.65	0.039	**
2(3H)-Furanone, 5-ethyldihydro-	1179	85	0.48	0.56	0.040	ns
2-Undecanone	1310	58	0.36	0.00	0.038	***
2(3H)-Furanone, dihydro-5-pentyl-	1400	85	0.00	0.36	0.037	***
**Total ketones**			47.48	45.81	10.626	ns

SEM: Standard error of the mean. Sig.: Significance. * (*p* < 0.05); ** (*p* < 0.01); *** (*p* < 0.001); ns: no significant difference.

**Table 5:** Effects of the production system on esters, ethers, furans and sulfur compounds (expressed as AU × 10^4^/g fresh weight) of BEDM lamb *longissimus thoracis et lumborum* muscle.

	LRI	*m*/*z*	Intensive	Extensive	SEM	Sig.
**Esters**						
Acetic acid, methyl ester	537	74	0.18	0.46	0.044	***
Ethyl Acetate	598	43	0.64	4.17	0.439	***
Formic acid, ethenyl ester	708	43	0.00	11.24	1.193	***
Butanoic acid, ethyl ester	856	70	1.51	0.00	0.158	***
Formic acid, heptyl ester	1062	56	0.00	5.69	0.599	***
Sulfurous acid, 2-ethylhexyl nonyl ester	1086	57	15.82	0.00	1.848	***
Formic acid, octyl ester	1147	55	0.00	3.78	0.431	***
Propanoic acid, 2-methyl-, 2-propenyl ester	1177	71	0.00	0.55	0.070	***
Butanoic acid, 2-propenyl ester	1183	71	0.00	0.63	0.080	***
2-Butenoic acid, 2-methyl-, 2-methylpropyl ester	1183	83	0.31	0.45	0.039	ns
2-Propenoic acid, 2-methyl-, (tetrahydro-2-furanyl)methyl ester	1297	71	1.00	0.00	0.103	***
Sulfurous acid, hexyl nonyl ester	1298	85	1.70	0.00	0.182	***
Sulfurous acid, 2-ethylhexyl hexyl ester	1331	85	0.00	0.45	0.049	***
Propanoic acid, 2-methyl-, 2-methylpropyl ester	1384	71	0.00	0.38	0.046	***
Sulfurous acid, 2-ethylhexyl isohexyl ester	1412	57	0.46	0.00	0.054	***
Pentanoic acid, 5-hydroxy-, 2,4-di-t-butylphenyl esters	1454	191	0.00	0.49	0.060	***
**Total esters**			21.62	28.30	1.509	*
**Ethers**						
Ether, 3-butenyl pentyl	1046	55	0.00	4.69	0.532	***
Ether, 2-ethylhexyl tert-butyl	1090	57	28.67	0.00	3.068	***
Decyl heptyl ether	1169	57	2.15	0.00	0.271	***
**Total ethers**			30.83	4.69	2.849	***
**Furans**						
Furan, 2-ethyl-	706	81	0.90	4.75	0.468	***
Furan, 2,3-dihydro-	806	70	0.00	1.76	0.228	***
2-n-Butyl furan	956	81	0.33	0.47	0.042	ns
Furan, 2-pentyl-	1054	81	17.49	6.41	1.331	***
**Total furans**			18.73	13.38	1.059	**
**Sulfur compounds**						
Dimethyl sulfide	528	62	0.43	1.67	0.211	**
Carbon disulfide	532	76	47.57	21.34	4.129	***
Dimethyl sulfone	1090	79	0.30	0.81	0.091	**
**Total sulfur compounds**			48.30	23.81	4.010	**

SEM: Standard error of the mean. Sig.: Significance. * (*p* < 0.05); ** (*p* < 0.01); *** (*p* < 0.001); ns: no significant difference.

## Data Availability

No new data were created or analyzed in this study. Data sharing is not applicable to this article.

## Supplementary Materials

The following are available online at https://www.mdpi.com/article/10.3390/foods10071450/s1, Table S1: Chemical composition, ingredients and amounts of mineral and vitamin mix used in the diet of intensively-reared lambs.
