# Supplementary material for: Influence of the Production System (Intensive vs. Extensive) at Farm Level on Proximate Composition and Volatile Compounds of Portuguese Lamb Meat
Source: Foods. 2021 Jun 22;10(7):1450. doi: 10.3390/foods10071450 (PMC8307059; doi:10.3390/foods10071450)
Supplement: Supplementary file 1 [file foods-10-01450-s001.zip › foods-1235643-supplementary.pdf]

**Supplementary Table S1:** Chemical composition, ingredients and amounts of mineral and vitamin mix used in the diet of intensively-reared lambs.

| **Nutrient** | **Commercial compound feed** |
|---|---|
| ***Chemical composition (%)*** | |
| Protein | 15.5 |
| Ash | 8.2 |
| Fat | 4.5 |
| Fibre | 8.2 |
| Calcium | 1.10 |
| Phosphorous | 0.4 |
| Sodium | 0.37 |
| ***Mineral Mix*** | |
| Fe (mg/kg) | 20 |
| Co (mg/kg) | 0.4 |
| Mg (mg/kg) | 62 |
| Zn (mg/kg) | 110 |
| Se (mg/kg) | 0.1 |
| I (mg/kg) | 0.6 |
| Cu (mg/kg) | 5 |
| ***Vitamin mix*** | |
| Vitamin A (UI/kg) | 8000 |
| Vitamin D3 (UI/kg) | 700 |
| Vitamin E (mg/kg) | 25 |

Commercial compound ingredients (in unknown proportions): Barley, wheat bran, extruded dehulled soy meal, dry beet pulp, brewers' dried grains, soy hulls, beet molasses, wheat germ, calcium carbonate, sunflower seed meal (extracted), soy oil, and sodium chloride.
